# 肿瘤微环境中肿瘤相关巨噬细胞极化的影响因素及其意义

**DOI:** 10.3779/j.issn.1009-3419.2023.106.07

**Published:** 2023-03-20

**Authors:** Wenke GE, Weibing WU

**Affiliations:** 210000 南京，南京医科大学第一附属医院胸外科; Department of Thoracic Surgery, The First Clinical Medical College of Nanjing Medical University, Nanjing 210000, China

**Keywords:** 肿瘤相关巨噬细胞, 极化, 肿瘤微环境, 肿瘤免疫, Tumor-associated macrophages, Polarization, Tumor microenvironment, Tumor immunity

## Abstract

肿瘤相关巨噬细胞（tumor-associated macrophages, TAMs）具有极高的可塑性，是肿瘤微环境（tumor microenvironment, TME）中含量最丰富的免疫细胞。各种TME成分（如细胞因子、趋化因子以及外泌体等）将TAMs招募到肿瘤区域内。随后，这些环境因子把TAMs诱导为抗肿瘤状态（M1样）或促肿瘤状态（M2样）之间的某一极化状态。另外，TAMs的极化过程是连续的，并且会随着肿瘤恶性进展而逐渐地朝向M2样状态，构成了一个有利于肿瘤生长和转移的正反馈环。因此，深入研究影响TAMs极化的各种因素和机制，有助于研发一种新的且可以与其他免疫治疗相结合的肺癌治疗策略。在既往的研究中已发现了许多可以促进TAMs的M2极化的分子和通路。然而，肿瘤细胞、间质细胞和TAMs之间这种复杂的串扰的潜在机制仍然难以研究清楚。在这篇综述中我们总结了促进TAMs向M2表型极化的各种因素，并进一步探讨了相关的分子机制。

## Abstract (en)

Tumor-associated macrophages (TAMs), characterized by high plasticity, are the most abundant immune cells in the tumor microenvironment (TME). TAMs are recruited to tumor region in response to various TME components such as cytokines, chemokines as well as exosomes. Subsequently, these environmental cues induce a certain polarization state of TAMs, ranging from anti-tumor states (M1-like) to pro-tumor states (M2-like). Furthermore, the polarization process of TAMs is continuous and gradually inclines toward the M2-like state with malignant progression, forming a positive feedback loop that facilitates tumor growth and metastasis. Consequently, figuring out the factors and mechanisms affecting TAMs polarization is beneficial for developing novel therapeutic strategies that can be combined with other immunotherapies for lung cancer. In previous studies, many significant molecules and pathways which could promote the M2 polarization of TAMs were identified. However, the underlying mechanisms of this sophisticated cross-talk between tumor cells, stroma cells and TAMs remain still difficult to by fully understand. In this review, we summarize the comprehensive factors involved in triggering TAMs polarization towards the M2 phenotype and further explore the relevant molecular mechanisms.

巨噬细胞是一群具有极高异质性的免疫细胞，具有不同的功能和表型，参与体内的固有免疫和适应性免疫^[1]^。在复杂的肿瘤微环境（tumor microenvironment, TME）的影响下，巨噬细胞可以被募集到肿瘤区域，并且被极化为抑制肿瘤生长的M1状态或促进肿瘤生长的M2状态^[2⇓-4]^。然而，一般我们所说的肿瘤相关巨噬细胞（tumor-associated macrophages, TAMs）表现为促进肿瘤恶化的M2表型。TAMs占TME中免疫细胞的大部分，包括组织驻留巨噬细胞（tissue-resident macrophages, TRMs）和被募集到肿瘤区域的骨髓源性巨噬细胞（bone marrow-derived macrophages, BMDMs）^[5,6]^。此外，TME中还有许多其他基质细胞和免疫细胞，比如肿瘤相关成纤维细胞（cancer-associated fibroblasts, CAFs），也在促进巨噬细胞极化方面中起到重要作用^[7,8]^。由于巨噬细胞有着极高的可塑性，其极化是一个连续的过程，因此我们有可能通过药物将M2极化状态逆转为M1状态，从而协助机体产生抗肿瘤效应^[9⇓-11]^。所以，研究TME中的哪些因子影响TAMs的极化，以及如何影响TAMs的极化，对于逆转巨噬细胞极化并抑制肿瘤生长的新治疗方法至关重要。本综述旨在阐述TAMs的生理作用，并探究肿瘤细胞、巨噬细胞和基质细胞复杂的相互作用，TME中的各种成分以及TAMs自身代谢对TAMs极化的影响。

## 1 TAMs的生理

### 1.1 TAMs的起源及其分类

在过去的研究^[12]^中一直认为TAMs只来源于骨髓释放的循环单核细胞，但新近研究^[13⇓⇓-16]^发现它们也可以来源于卵黄囊和胎儿肝脏的红髓系祖细胞，这些巨噬细胞可以在局部器官发育为组织驻留巨噬细胞，成为TAMs的来源之一，并且这些细胞可以在身体局部通过自我更新来维持数量稳定^[17⇓-19]^。所以在TME中这两种不同来源的巨噬细胞都可以被极化，并获得相关表型。但是不同来源的巨噬细胞往往聚集在肿瘤的不同区域，循环单核细胞来源的TAMs往往聚集在肿瘤的边缘，而胚胎来源的TAMs大多数聚集在肿瘤核心区域^[20]^，这提示这两种巨噬细胞对肿瘤的影响或许是不同的。

在巨噬细胞分类方面，Mills^[21]^首次提出了根据其功能将巨噬细胞分为经典激活的巨噬细胞（M1）和替代激活的巨噬细胞（M2）。TAMs的M1表型可由干扰素γ（interferon gamma, IFN-γ）、Toll样受体（Toll-like receptor, TLR）的激动或集落刺激因子2（colony stimulating factor 2, CSF2）等诱导而来，并且表达大量促炎因子来发挥抗肿瘤效应，比如诱导型一氧化氮合酶（inducible nitric oxide synthase, iNOS）、活性氧（reactive oxygen species, ROS）、白细胞介素（interleukin, IL）-12和IL-23等^[22]^；相反，IL-4和IL-13可促进TAMs的M2表型，使之表达高水平的IL-10和转化生长因子-β（transforming growth factor-β, TGF-β）等有着促进肿瘤生长效应的相关因子，这些因子不仅可以诱发免疫抑制、血管生成、肿瘤生长和转移，还可以导致对检查点抑制剂或过继性T细胞免疫治疗耐药^[2,4,22]^。然而，随着对TAMs标志物和单细胞测序技术的进一步研究，这种M1/M2分类方式的主导地位正在动摇。研究^[23]^显示，由于TAMs的极化是一个连续的过程，M1或M2只代表了极化过程的两个极端，而TAMs可以同时表达M1和M2两者的生物学标记，并同时具有M1抗炎和M2促炎的特性，这给我们对巨噬细胞极化机制的研究带来困难，所以这种M1/M2的二分法太过于简单以至于不再适用于巨噬细胞分类^[24,25]^。现在，有学者基于TAMs在TME下的不同刺激物和功能，将TAMs的M2极化状态进一步细分^[26,27]^。尽管该分类方法比之前的M1/M2学说更细致，但是在理论上，由于TAMs的极高的异质性和可塑性，它们的极化亚群数量应该非常多。这种新分类方式的复杂性使得对于最合适的TAMs分类学说仍需进一步研究。但如果研究人员仅关注TAMs的抗肿瘤状态或促肿瘤状态的趋势，采用M1和M2极化状态这种简单的分类方式已经足够。

### 1.2 TAMs的功能

TAMs的极化往往与肿瘤进展息息相关，其M1和M2状态对肿瘤产生明显相反的作用。这种功能的差异部分取决于这两类巨噬细胞对精氨酸的代谢途径。M1表型TAMs通过iNOS将精氨酸分解为一氧化氮和瓜氨酸，而NO与巨噬细胞的细胞毒性和抗肿瘤效应密切相关；M2表型TAMs由精氨酸酶1（arginase 1, Arg1）将精氨酸水解成鸟氨酸和尿素，而精氨酸的减少则会影响T细胞和自然杀伤（natural killer, NK）细胞的活化和增殖，进而引发机体的免疫抑制^[21,28]^。因此，在理论上，或许将促进肿瘤恶化的M2型TAMs转变为杀伤肿瘤的M1型TAMs可以通过刺激自身固有免疫达到抗肿瘤的目的。

此外，M1型TAMs不仅可以表达大量促炎因子如肿瘤坏死因子（tumour necrosis factor α, TNF-α）、IL-1β、IL-6、IL-12和IL-23来促进Th1（细胞毒性）型反应抑制肿瘤^[29]^，还可以上调抗原的加工和呈递相关基因、提高共刺激分子和主要组织相容性复合体II类（major histocompatibility complex-II, MHC-II）的表达来促进T细胞活化杀灭肿瘤^[11]^。值得考虑的是，大量的M1型巨噬细胞虽然在杀伤肿瘤细胞方面有着不可或缺的作用，但是其释放过多这种促炎因子是否会导致全身炎症反应综合征等其他不良后果还有待进一步探究。相反，M2型TAMs主要通过诱发免疫抑制、促进肿瘤生长和转移、促进血管生成等多方面加速癌症恶化。除了通过上述精氨酸酶代谢的方法外，M2型TAMs还可以通过在缺氧的微环境下募集FOXP3^+^调节性T细胞来阻碍T细胞的抗肿瘤效应、促进形成免疫抑制的TME^[16,30]^。此外，在免疫抑制的微环境下，M2状态的TAMs还可以分泌Th2型细胞因子，例如IL-4、IL-13、IL-10以及表皮生长因子（epidermal growth factor, EGF）、血小板源性生长因子（platelet-derived growth factor, PDGF）、TGF-β、IL-8、血管内皮生长因子A（vascular endothelial growth factor A, VEGFA）、碱性成纤维细胞生长因子（basic fibroblast growth factor, BFGF）等，这些因子不仅可以刺激肿瘤产生和增殖^[31⇓-33]^，还可以促进肿瘤区域血管的生成，为肿瘤提供丰富的血供和营养来帮助肿瘤生长和播散^[34⇓-36]^。另外，在肿瘤转移方面，M2极化状态的巨噬细胞可以分泌大量包括基质金属蛋白酶（matrix metalloproteinases, MMPs）和组织蛋白酶在内的多种可以降解细胞外基质、基底膜的酶，以此来协助肿瘤远处转移^[37⇓-39]^。由此可见，M2型TAMs与癌症的多种恶性事件息息相关，它们不仅可以通过释放各种促瘤因子帮助肿瘤生长、转移，还可以通过调节其他免疫细胞的功能进一步形成有利于肿瘤发生发展的免疫抑制微环境。

TAMs的极化状态不是一成不变的，而是连续、动态变化的。一般情况下，在肿瘤进展的不同阶段，TAMs的极化状态往往由M1型转变为M2型。在早期的肿瘤中，M1型TAMs占大多数，并且承担着杀灭肿瘤细胞和激活免疫反应的作用。但在另一方面，M1型TAMs也会导致慢性炎症，进而使癌细胞中基因组更加不稳定，促进肿瘤进展^[40]^。随着癌症恶化，TAMs的表型会在TME的影响下由杀伤肿瘤的M1表型转化为促进肿瘤进展的M2表型，M2表型的TAMs的相对数量逐渐增多，进一步导致肿瘤恶化^[3]^。因此，TAMs的促肿瘤状态极化会形成有利于肿瘤生长的正反馈，对各种临床治疗的疗效有着极大的阻碍，这促使我们加强对逆转TAMs极化状态的相关研究。

## 2 TME中影响TAMs极化的因素

TAMs的极化往往指的是其在某一特定时间点的激活状态^[3]^，而且在肿瘤进展的过程中，由于TAMs有着高度的可塑性和异质性，TAMs可在缺氧、TME中的细胞因子或外泌体等因素的作用下，出现了极化状态转向M2的变化^[4]^（图1）。研究^[40,41]^已经证实M2型TAMs的相对数量增多与癌症患者较差的5年生存率密切相关。因此，探究在肿瘤免疫微环境中影响巨噬细胞M2极化状态的各种因素尤为重要，并有利于改善M2型巨噬细胞增多型癌症患者的疗效和提高该类癌症患者对免疫治疗的反应。

**图1:**
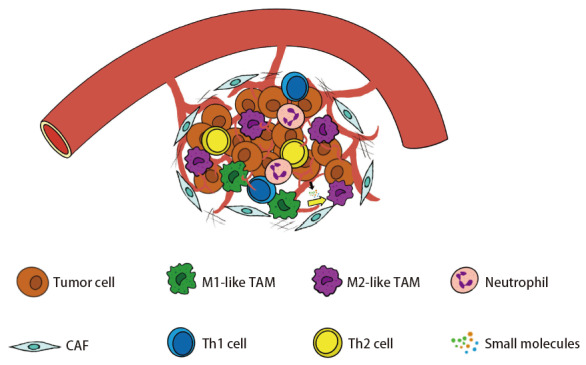
肿瘤微环境下的TAMs极化。 在肿瘤区域，肿瘤细胞和其他基质细胞、免疫细胞以及环境中的各种小分子物质，比如肿瘤相关成纤维细胞、肿瘤相关巨噬细胞、细胞因子等，共同组成了肿瘤微环境。在肿瘤微环境的诱导下，抑制肿瘤的M1型TAMs被极化为促肿瘤的M2型TAMs。

### 2.1 缺氧

肿瘤细胞的快速增殖特性往往造成了TME中供氧和耗氧之间的不平衡，导致微环境缺氧，再者，人体正常分化的细胞通常依赖氧气在线粒体中进行氧化磷酸化为其提供能量，而肿瘤细胞即使在氧充足的情况下也通过无氧糖酵解来为其快速增殖供能，并产生大量乳酸，这也被成为“瓦博格效应（Warburg effect）”^[42,43]^。因此，随着大量乳酸的产生，TME的pH值越来越低，这干扰了T细胞和NK细胞的抗肿瘤功能^[43]^，并且肿瘤细胞产生的乳酸还能通过缺氧诱导因子-1α（hypoxia-inducible factor-1α, HIF-1α）增强VEGF、Arg1和其他M2相关基因的表达来促进TAMs的M2极化，而且研究^[44]^发现这种现象在HIF-1α缺乏的巨噬细胞中不能发生，充分证明了乳酸和HIF-1α在缺氧条件下促进巨噬细胞M2极化的重要作用。另外，研究^[45]^发现在缺氧条件下的胰腺导管腺癌微环境中，表达大量HIF-2的CAFs可以促进TAMs的M2极化，并将其Arg1的表达水平提高约4倍。相反，采用HIF-2抑制剂PT2399处理CAFs可以显著地减弱TAMs的M2极化^[46]^，这提示了缺氧的CAFs可以通过依赖HIF-2的方式促进TAMs的M2极化。此外，研究人员推测CAFs分泌了一些HIF-2依赖性的可溶性分子来介导TAMs的M2极化^[45,46]^，然而，具体是哪些分子和通路影响了此过程还需要进一步探索。

### 2.2 细胞因子和趋化因子

细胞因子和趋化因子是免疫细胞和非免疫细胞分泌的生物活性小分子蛋白质，在TME中可以通过控制TAMs的极化来调控免疫应答^[27]^（表1）。以往的研究^[47]^已经证实细胞因子粒-巨噬细胞集落刺激因子（granulocyte-macrophage colony-stimulating factor, GM-CSF/CSF2）和巨噬细胞定殖刺激因子（macrophage colony-stimulating factor, M-CSF/CSF1）可以分别通过干扰素调节因子5（interferon regulatory factor-5, IRF5）和IRF4诱导TAMs的M1和M2极化状态。此外，肺癌细胞可通过表达大量Oct4，促进自身细胞分泌M-CSF，从而促进TAMs的M2极化。另外，具有促炎特性的M1型巨噬细胞也可以增强肺癌细胞中Oct4的表达，并且与M2极化紧密相关^[48]^。使用伊马替尼阻断集落刺激因子受体，可以通过抑制信号传导及转录激活蛋白6（signal transducer and activator of transcription 6, STAT6）磷酸化和核易位来阻断TAMs的M2极化^[49]^，充分证实了该细胞因子重塑TAM表型的重要作用。

**表1:** 细胞因子和趋化因子对TAMs极化状态的影响

Molecule	Signal pathway	Effect
M-CSF/CSF1	PLC-γ2/STAT3/ERK1/2	M2
IL-4/IL-13	STAT6	M2
IL-6	ERK/STAT6	M2
IL-10	CaMKII/ERK1/2/STAT3	M2
TGF-β	Smad2/3/c-Myc/CXCR4/STAT3	M2
CXCL12	PTEN/PI3K/PKB	M2
GM-CSF/CSF2	JAK2-STAT5/ERK/PKB/NF-κB/IRF5	M1
TNF-α	NF-κB	M1
IFN-γ	STAT1	M1

M-CSF/CSF1: macrophage colony-stimulating factor; IL: interleukin; TGF-β: transforming growth factor-β; CXCL: C-X-C motif chemokine ligand; GM-CSF/CSF2: granulocyte-macrophage colony-stimulating factor; TNF-α: tumour necrosis factor-α; IFN-γ: interferon gamma; PLC-γ2: phospholipase C-γ2; STAT: signal transducer and activator of transcription; ERK: extracellular regulated protein kinases; CXCR: C-X-C motif chemokine receptor; CaMKII: calmodulin-dependent protein kinase II; PTEN: phosphatase and tensin homolog; PI3K: phosphatidylinositol 3-kinase; PKB: protein kinase B; JAK2: Janus kinase 2; NF-κB: nuclear factor kappa B; IRF5: interferon regulatory factor 5.

另外，在缺氧条件下，IL-6激活了细胞外调节蛋白激酶（extracellular regulated protein kinases, ERK）信号通路调控，不仅提高了CD209^+^和CD206^+^（M2巨噬细胞标记物）细胞的比例，而且还提高了Arg1和YM1等与M2极化状态相关的mRNA表达^[50]^。此外，研究^[51]^证实，M2样TAMs中大量表达的Wnt5a可以增加这些细胞中IL-10的分泌，而IL-10又可作为自分泌细胞因子通过钙调蛋白依赖性蛋白激酶II（calmodulin-dependent protein kinase II, CaMKII）-ERK1/2-STAT3通路促使这些TAMs的M2极化；同时，用IL-10抗体处理可明显阻断M2极化，表明IL-10在调节TAMs的极化状态中发挥了重要作用。TGF-β作为TME中重要的调节免疫的细胞因子，也在前列腺腺癌的模型下被证实有控制M2极化状态的效应。因为抑癌基因肿瘤高甲基化基因1（hypermethylated in cancer 1, HIC1）与TGF-β表达量呈负相关，所以前列腺癌细胞中HIC1的高甲基化或缺失则会导致基质中TGF-β表达升高，并且TGF-β通过激活STAT3信号通路、增强由c-Myc通路调控的CXC受体4（C-X-C motif chemokine receptor 4, CXCR4）表达，进一步促进巨噬细胞M2状态^[52]^。TGF-β受体I抑制剂Galunisertib可以用来抑制该效应，证明了在前列腺癌细胞中HIC1/TGF-β轴可以调控TAM极化进而形成免疫抑制的TME^[52]^。

趋化因子是一种小分子蛋白质，在多种免疫细胞的运输和分化中起到重要作用。研究人员^[53,54]^根据趋化因子氨基端（N端）半胱氨酸的排列方式，将其分为四个亚族：CXC、CC、XC和CX3C。在结直肠癌中，趋化因子CXCR4的表达增多不仅有利于募集巨噬细胞到肿瘤区域，还可促进趋化因子CXC配体12（C-X-C motif chemokine ligand 12, CXCL12）/CXCR4轴激活并携带大量miR-25-3p、miR-130b-3p和miR-425-5p的外泌体分泌并传递到TAMs，这些miRNA通过磷酸酯酶与张力蛋白同源物（phosphatase and tensin homolog, PTEN）/磷脂酰肌醇3-激酶（phosphatidylinositol 3-kinase, PI3K）/蛋白激酶B（protein kinase B, PKB）信号通路促进TAMs的极化状态向M2表型转变^[55]^。另外，趋化因子CCL2和CCL5也被证实与M2样巨噬细胞标记物（甘露糖受体C1样蛋白1和IL-10）的表达呈正相关，使用携带有编码双特异性结合并中和CCL2和CCL5的特异性mRNA可以同时抑制这两种趋化因子的功能，将TAMs的极化状态由M2逆转为M1，提示趋化因子CCL2和CCL5可作为免疫调节器促进TAMs的M2极化^[56]^。这些细胞因子和趋化因子在TME中介导了肿瘤细胞与TAMs之间的“串扰（cross-talk）”。然而，由于细胞因子和趋化因子种类繁多，数量巨大，通过这些因子来达到逆转TAMs极化的结果或许是行不通的，并且在治疗中有可能导致Th1型和Th2型细胞因子的失衡，造成其他严重的全身不良反应。

### 2.3 代谢重塑

M1和M2极化状态的TAMs具有不同的代谢模式，并通过不同的代谢途径补给能量。研究^[22,57]^表明，具有杀伤肿瘤特性的M1表型TAMs通常利用糖酵解代谢来提供细胞所需的能量，而免疫抑制的M2表型TAMs则优先通过氧化代谢途径如氧化磷酸化（oxidative phosphorylation, OXPHOS）和脂肪酸氧化（fatty acid oxidation, FAO）来提供能量。因此，增强脂质分解代谢对TAMs的M2极化十分重要^[58]^。研究^[59]^已经证明，TME中的脂质积累对TAMs的极化至关重要，TAMs通过增加清道夫受体CD36的表达来摄取肿瘤细胞中更多的脂质，导致脂质在TAMs中不断积累，并增强了TAMs中的脂肪酸氧化和氧化磷酸化，并通过激活STAT6信号通路将TAMs极化为M2状态。

此外，其他研究^[60]^表明，肿瘤来源的胞外脂质还可以诱导TAMs中PI3K-γ表达的上调，并抑制对核因子κB（nuclear factor-κB, NF-κB）p65磷酸化，从而通过PI3K-γ信号通路促使TAMs极化为M2表型。研究^[61]^还发现，受体相互作用蛋白4激酶3（receptor-interacting protein 4 kinase 3, RIPK3）可以调节TAMs中的脂肪酸代谢，并进而影响TAMs的极化状态。在肝癌发生过程中受TME中因子的影响，肿瘤区域内TAMs中RIPK3的表达逐渐降低，这会激活TAMs中过氧化物酶体增殖物激活受体（peroxisome proliferators-activated receptors, PPAR）通路并增强这些细胞的脂肪酸代谢，从而促进其M2极化状态；相反，使用地西他滨可以导致RIPK3的低甲基化解除其沉默作用，上调了该激酶的表达水平并降低了PPARα和PPARγ的表达，进而将TAMs的极化状态由M2状态逆转为M1^[61]^。所以，脂质的氧化可以通过多种通路促进TAMs的促肿瘤表型。

另外，TAMs对脂质的摄取也非常重要。除了上述提及的CD36，脂滴（lipid droplets, LDs）也作为TAMs稳定的脂肪酸来源。LDs可通过哺乳动物雷帕霉素靶蛋白（mammalian target of rapamycin, mTORC）信号通路促进TAMs线粒体呼吸，从而使M2样TAMs的相关基因（Mrc1, Arg1, Retnla, Chil3, VEGFA, MMP9）表达增多和精氨酸酶活性增强，如果采用相应的抑制剂阻断LDs的形成或降解则会减弱巨噬细胞的M2极化^[58]^。不仅TAMs内部脂质氧化的主动增强可以促进M2极化，TAMs细胞膜内脂质的被动流失依然会导致此现象。例如，卵巢癌细胞可分泌透明质酸，它作为TME中重要的细胞外基质可以促使TAMs细胞膜中胆固醇的流出。这种膜胆固醇的流失明显增加了巨噬细胞M2相关基因的表达，同时抑制了M1相关基因的表达，并通过激活STAT6和PI3K驱动TAMs极化为M2表型。研究^[62]^发现使TAMs中ATP结合盒式蛋白（ATP-binding cassette transporter, ABC transporter）的基因缺失来抑制胆固醇外排可以抑制这种效应，说明了TAMs细胞膜中胆固醇的流失对其M2极化的促进作用。

然而，尽管很多研究^[58⇓⇓-61]^认为TAMs的脂肪酸氧化加强与其M2极化有着密切关联，但也有研究^[63]^发现，TAMs脂肪酸氧化的减弱仍可能促进其M2表型。例如，含半胱氨酸的天冬氨酸蛋白水解酶-1（cysteinyl aspartate specific proteinase-1, caspase-1）通过在Asp64处切割PPARγ，从而产生一个41 kDa的片段。这个被截断的PPARγ然后又易位到线粒体，直接抑制中链酰基辅酶a脱氢酶活性并且抑制了TAMs脂肪酸的氧化，导致脂滴积累，进而诱导TAMs促肿瘤表型；除此之外，TAMs线粒体中的中链酰基辅酶a脱氢酶活性的降低还可以导致TAMs分泌乳酸增多，造成TME中pH值的降低^[63]^。正如前文所提到，微环境中的乳酸增多可能会通过抑制其他免疫细胞的功能，进一步促进M1型TAMs向M2型极化，创造有利于肿瘤生长的免疫抑制环境^[43,44]^。因此，针对脂肪酸氧化对TAMs极化状态的影响，不同的研究人员持有不同的观点，这可能是由于不同的实验条件或实验环境造成的，需要进一步深入研究。

### 2.4 外泌体

肿瘤细胞与免疫细胞、基质细胞等相互之间存在着精密而复杂的相互作用，这种“串扰”往往需要一些物质（如外泌体）使各细胞之间的联系更加紧密^[64]^。外泌体是一种源自核内体的细胞外囊泡，它们可以携带蛋白质、脂质、DNA、RNA以及代谢物等分子，并将这些物质运输给其他细胞来调节细胞间的通讯^[65]^。此外，外泌体不仅在缺氧环境中促进巨噬细胞M2极化，而且在正常氧气环境下，它们也可以通过向巨噬细胞输送多种分子来发挥这种作用^[65⇓-67]^。

研究^[68]^已经证实，结直肠癌细胞可以通过hnRNPA2B1介导将外泌体miR-934传递到骨髓源性巨噬细胞中，这种miRNA抑制PTEN的表达，从而激活PI3K/PKB信号通路，导致M2标记物（CD163、CD206、Arg1和IL-10）的表达增加；同时，M2极化状态的TAMs分泌CXCL13来诱导转移前生态位的形成并促进结直肠癌肝转移，同时形成了结直肠癌细胞中的CXCL13/CXCR5/NF-κB/p65/miR-934正反馈环，进一步促进肿瘤转移和巨噬细胞M2极化。此外，肿瘤细胞释放的外泌体miRNA的减少也会影响TAMs的极化。研究^[69]^显示，SHH型髓母细胞瘤表达较低水平的外泌体let-7i-5p和miR-221-3p并被TAMs内化，然后激活TAMs中的PPARγ来促进其极化向着M2表型；但是，抑制外泌体的分泌和摄取却会减弱M2极化，这背后的具体机制还需要进一步研究。此外，在缺氧的TME中，外泌体在控制TAMs极化状态方面也发挥着重要作用，研究^[66]^发现，胰腺癌细胞在缺氧的条件下可以依赖HIF-1α和HIF-2α途径将大量携带miR-301a-3p的外泌体运输给TAMs，而miR-301a-3p又激活了TAMs中PI3Kγ信号通路并下调抑癌基因PTEN的表达，从而使TAMs转换为M2表型。

除了肿瘤细胞以外，内皮细胞也可以释放含有多种miRNA的外泌体调控TME中肿瘤细胞、内皮细胞和TAMs之间的“串扰”。研究^[70]^已经证实，内皮细胞在肿瘤区域被激活并释放携带miR-142-5p、miR-183-5p和miR-222-3p的外泌体，这些miRNA将巨噬细胞募集到肿瘤区域，并通过抑制PTEN的表达使它们极化为促肿瘤状态。在不同的肿瘤中，长链非编码RNA（long noncoding RNA, lncRNA）也能通过外泌体发挥调节TAM极化的功能，在缺氧条件下，肿瘤细胞还分泌一种致癌的lncRNA，即小核仁RNA宿主基因7（small nucleolar RNA host gene 7, SNHG7），lncRNA SNHG7被分泌后装配入外泌体中，招募cullin 4A并促进PTEN泛素化，从而激活PI3K/PKB通路，最终促进了TAMs的M2极化，并导致了肺腺癌细胞对多西他赛类抗肿瘤药物产生抗药性^[71]^。

外泌体作为TME中肿瘤细胞与其他细胞相互交流的“通讯兵”，在TAMs的极化过程中发挥重要作用。然而，使用缺乏特异性的外泌体抑制剂处理肿瘤细胞以及TAMs，往往会导致细胞之间的通讯都受到干扰，同时也不能有效地促进TAMs由M2到M1极化状态的转化。尽管如此，我们或许可以干扰外泌体中miRNA或lncRNA的含量，并通过外泌体将它们转运到TAMs中，从而发挥相应作用。

### 2.5 肿瘤相关成纤维细胞

在TME中CAFs与TAMs之间存在密切联系，这两种细胞通常相互作用，促进癌症的进展^[72]^。CAFs可以通过多种途径诱导巨噬细胞促肿瘤的极化，导致M2标记物如CD68和CD163的升高。此外，正常结肠中的成纤维细胞也可以促进巨噬细胞极化向M2转换^[73]^，研究人员认为CAFs也许保留了结肠中正常成纤维细胞的一些内在功能，如将巨噬细胞极化为抗炎状态，但在TME的影响下，CAFs同时获得了其他促进肿瘤恶化的功能，如干扰免疫治疗的疗效、与TAMs协同调节免疫抑制等。

在复发性骨肉瘤中，CAFs分泌赖氨酰氧化酶（lysyloxidase, LOX）参与上皮间质转化（epithelial-mesenchymal transition, EMT）并重塑肿瘤免疫微环境，且该酶与M2巨噬细胞极化状态高度相关。用特异性不可逆LOX抑制剂β-氨基丙腈处理后，在体内和体外实验中，M2巨噬细胞的相对数量都显著降低^[7]^。然而，这种效应背后的详细机制还未被阐明。值得思考的是，在过去的研究^[74]^中有学者对LOX在骨肉瘤中的作用持有不同的观点，他们认为LOX表达的增多不仅抑制了骨肉瘤的快速增殖和转移，而且还促进肿瘤细胞凋亡，这提示LOX在人骨肉瘤中是肿瘤抑制因子，而不是致癌因子。因此，LOX调控巨噬细胞的具体机制需要进一步探究。另外，部分的CAFs来源于内皮细胞的内皮细胞向间充质转化（endothelial to mesenchymal transition, EndoMT）^[75]^，在胰腺导管腺癌中，EndoMT细胞可以分泌细胞伴侣热休克蛋白90α（heat shock protein 90α, HSP90α）。这些胞外的HSP90α可以与TAMs上的受体TLR4和CD91结合，导致了M2相关标记物CD163、CD204、IL-10、TGF-β及代谢标记物Arg1基因的表达增强，以及M1标记物的表达下调，而使用HSP90α抗体处理巨噬细胞可以阻碍这个现象^[8]^，这些数据提示了CAFs分泌的LOX以及HSP90α在促进TAMs的M2极化方面起到了重要作用，然而，尽管研究人员针对CAFs对TAMs极化的影响做了许多研究^[72⇓⇓-75]^，但是，利用CAFs将M2型TAMs极化为M1型的相关研究有待发掘，同时，CAFs与TAMs之间串扰的具体调节机制也有待进一步探索。

## 3 总结与展望

巨噬细胞作为TME中主要组成部分之一，可以与肿瘤细胞、基质细胞和免疫细胞相互作用，形成了一个复杂的信息网络，塑造了一个有利于肿瘤生长的免疫抑制微环境^[76]^。本综述的重点在于讨论TME中的多种因素、CAFs、外泌体和TAMs自身代谢对TAMs极化状态的影响。由于TAMs有着极强的异质性和可塑性，它们会根据TME中各种细胞因子、趋化因子、小分子活性物质的改变而转变极化状态。M2表型的TAMs与TME中免疫抑制和免疫治疗的耐药有着重要联系，研究人员可采用各种治疗方法，比如携带药物的纳米粒子，促使巨噬细胞重编程，将巨噬细胞从促肿瘤的M2极化状态复极化为抗肿瘤的M1状态，以辅助其他类型免疫治疗，提高肺癌的综合治疗效果^[27,77]^。但是，针对TAMs治疗所需要的最佳给药剂量和频率是很难确定的，因为在停止使用促使TAMs复极的药物之后，TAMs的表型和功能可能恢复到之前的M2样免疫抑制状态^[78]^。况且，决定巨噬细胞的最终极化状态需要微环境内多种调节因子以及TAMs自身多种代谢通路的共同调节，这些因素导致目前靶向巨噬细胞极化的药物具有各种局限性，以及撤药后的疗效无法维持^[11]^。然而，相较于直接阻碍TAMs的募集或减少TAMs的数量这两种靶向TAMs的治疗方法，将其复极化更新颖且更具安全性，这需要进一步研究TAMs极化背后的具体机制。此外，在各种复杂的TAMs极化机制中，如何挑选出合适的通路以确保肺癌综合治疗的有效性和安全性以及挽救更多失去手术机会的肺癌患者，也是需要研究的方向。
